# TNFα-Induced Inflammation Model—Evaluation of Concentration and Passage-Dependent Effects on Bovine Chondrocytes

**DOI:** 10.3390/ijms25179136

**Published:** 2024-08-23

**Authors:** Robert Ossendorff, Su Wang, Sarah Kurth, Max Jaenisch, Elio Assaf, Andreas C. Strauss, Damien Bertheloot, Kristian Welle, Christof Burger, Dieter C. Wirtz, Frank A. Schildberg

**Affiliations:** Department of Orthopedics and Trauma Surgery, University Hospital Bonn, 53127 Bonn, Germany

**Keywords:** TNFα, inflammation model, cartilage, chondrocyte, cytokine, bovine

## Abstract

Inflammation models are widely used in the in vitro investigation of new therapeutic approaches for osteoarthritis. TNFα (tumor necrosis factor alpha) plays an important role in the inflammatory process. Current inflammation models lack uniformity and make comparisons difficult. Therefore, this study aimed to systematically investigate whether the effects of TNFα are concentration-dependent and whether chondrocyte expansion has an effect on the inflammatory model. Bovine chondrocytes were enzymatically isolated, expanded to passages 1–3, and transferred into a 3D pellet culture. Chondrocyte pellets were stimulated with recombinant bovine TNFα at different concentrations for 48 h to induce inflammation. Gene expression of anabolic (*collagen 2*, *aggrecan*, *cartilage oligomeric protein* (*COMP*)), catabolic (matrix metalloproteinases (*MMP3*, *MMP13*)), dedifferentiation (*collagen 1*) markers, inflammation markers (*interleukin-6* (*IL-6*), *nuclear factor kappa B* (*NFkB*), *cyclooxygenase-2* (*COX*), *prostaglandin-E-synthase-2* (*PTGES2*)), and the apoptosis marker *caspase 3* was determined. At the protein level, concentrations of IL-6, nitric oxide (NO), and sulfated glycosaminoglycans (GAG) were evaluated. Statistical analysis was performed using the independent t-test, and significance was defined as *p* < 0.05. In general, TNFα caused a decrease in anabolic markers and an increase in the expression of catabolic and inflammatory markers. There was a concentration-dependent threshold of 10 ng/mL to induce significant inflammatory effects. Most of the markers analyzed showed TNFα concentration-dependent effects (*COMP*, *PRG4*, *AGN*, *Col1*, *MMP3*, and *NFkB*). There was a statistical influence of selected gene expression markers from different passages on the TNFα chondrocyte inflammation model, including *Col2*, *MMP13*, *IL-6*, *NFkB*, *COX2,* and *PTGES2*. Considering the expression of *collagen 2* and *MMP3*, passage 3 chondrocytes showed a higher sensitivity to TNFα stimulation compared to passages 1 and 2. On the other hand, *MMP13*, *IL-6*, *NFkB,* and *caspase 3* gene expression were lower in P3 chondrocytes compared to the other passages. On the protein level, inflammatory effects showed a similar pattern, with cytokine effects starting at 10 ng/mL and differences between the passages. TNFα had a detrimental effect on cartilage, with a clear threshold observed at 10 ng/mL. Although TNFα effects showed concentration-dependent patterns, this was not consistent for all markers. The selected passage showed a clear influence, especially on inflammation markers. Further experiments were warranted to explore the effects of TNFα concentration and passage in long-term stimulation.

## 1. Introduction

Osteoarthritis (OA) is a chronic disease that affects the whole joint, involving all joint tissues, including cartilage, bone, menisci, adjacent synovial structures, and the infrapatellar fat pad [1,2,3]. OA produces enormous financial costs to the individual and the health care system due to its high prevalence in society [4]. Under normal conditions, the interaction between anabolic and catabolic processes in the knee joint maintains a delicate balance. However, in OA, this balance is disrupted, leading to deterioration of articular cartilage function and subsequent joint damage [5]. While the exact causes and mechanisms behind this debilitating condition remain unclear, inflammatory molecules such as proinflammatory cytokines are believed to play a critical role in the dysregulated processes associated with the pathophysiology of OA [6]. These proinflammatory factors can inhibit cartilage matrix synthesis and promote its degradation by increasing the production of catabolic matrix metalloproteinases (*MMPs*) while suppressing the synthesis of extracellular matrix components such as proteoglycan and collagen, thus contributing to cartilage degradation [7,8,9,10]. Tumor necrosis factor α (TNFα) plays a critical role in this process by driving inflammatory cascades and cartilage degradation, as its stimulation of numerous other cytokines and enzymes activates a proinflammatory cycle [9,11,12,13,14]. Inflammation models of OA using proinflammatory cytokines such as TNFα have been established to investigate the pathological mechanisms and evaluate the efficacy of novel drugs or therapies [15,16,17]. Other inflammation chondrocyte models are performed with IL-1ß, IL-6, IFN-γ, LPS, synovial fluid (SF) from OA patients, or conditioned medium from other cells [18,19].

While many studies have employed 2D models, it is important to recognize that cartilage is inherently a 3D structure in which chondrocytes reside within an extracellular matrix composed mainly of proteoglycan and collagen [20]. Cultivation in 3D culture systems induces re-differentiation of the chondrocyte phenotype and maintains cell–cell and cell–matrix interactions that are important for the study of cartilage [21,22,23]. In vitro expansion of chondrocytes is usually required to obtain sufficient numbers of chondrocytes. During the expansion phase, these cells undergo dedifferentiation, where they can lose the chondrocyte-specific phenotype and instead express a fibroblast-like shape [24].

Given the variability of available in vitro and ex vivo OA/inflammation models across studies, including different model species, cell culture or tissue explants, cytokine concentrations, cell expansion procedures, and duration of administration, comparisons between studies are challenging [18,25]. In this study, we used a standardized bovine chondrocyte inflammation model with the proinflammatory cytokine TNFα to investigate: (1) whether the inflammatory effect induced by TNFα is concentration-dependent, and (2) whether in vitro expansion of chondrocytes has an effect on the inflammation model.

## 2. Results

### 2.1. Anabolic Markers

In general, TNFα supplementation resulted in a decrease in anabolic markers at the gene expression level of chondrocytes in 48-h pellet culture. The anti-anabolic effect was influenced by the concentration of TNFα. Passages showed differences in the sensitivity of TNFα-induced effects.

**Figure 1:**
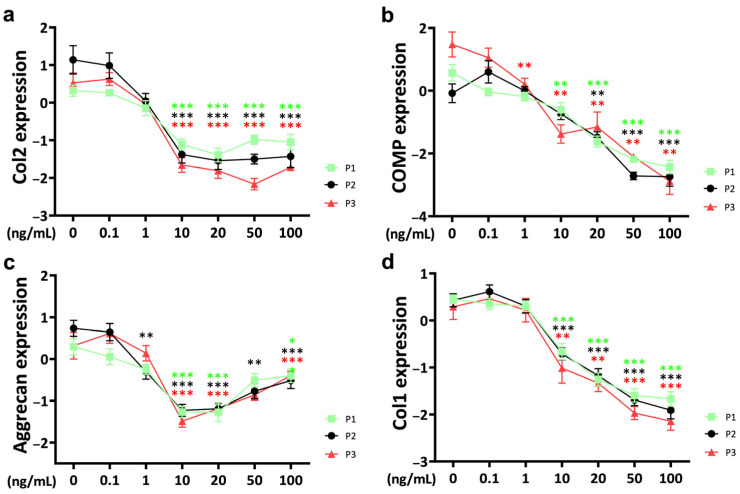
Gene expression of anabolic markers (**a**) *collagen 2* (*Col2*), (**b**) *cartilage oligomeric protein* (*COMP*), (**c**) aggrecan and (**d**) *collagen 1* (*Col1*) from different passages treated with different doses (0.1 ng/mL, 1 ng/mL, 10 ng/mL, 20 ng/mL, 50 ng/mL, and 100 ng/mL) of TNFα for 48 h in a bovine chondrocyte pellet culture model. Gene expression data were normalized to day 0 and natural log transformed. * *p* < 0.05; ** *p* < 0.01; *** *p* < 0.001. The green, black, and red stars represent comparisons with the control group in passage 1, passage 2, and passage 3, respectively. Figures show only results compared within the same passage. P1: passage 1; P2: passage 2; P3: passage 3; *Col2*: *collagen 2*; *Col1*: *collagen 1*; *COMP*: *cartilage oligomeric protein*.

In this study, TNFα supplementation resulted in a downregulation of *collagen 2* (*Col2*) expression (Figure 1a). Notably, a significant difference from the control occurred at a threshold concentration of 10 ng/mL. Concentrations higher than 10 ng/mL of TNFα did not show a stronger effect. Cytokine effects on chondrocyte gene expression of *Col2* in chondrocytes showed differences between passages. At 0.1 ng/mL, *Col2* expression was higher in passage 3 compared to passage 1 (*p* = 0.039). However, when the TNFα concentration reached 10 ng/mL, this pattern was reversed, and *Col2* gene expression was lower in passage 3 (*p* = 0.027) compared to passage 1. This trend persisted with further increases in concentration; *Col2* expression remained consistently lower in passage 3, even at TNFα concentrations as high as 100 ng/mL (*p* = 0.023).

Chondrocyte gene expression of *cartilage oligomeric protein* (*COMP*) was negatively influenced by TNFα starting at 1 ng/mL in passage 3 (*p* = 0.005) (Figure 1b). At 10 ng/mL, a significant decrease in *COMP* gene expression was observed in chondrocytes from passage 1 (*p* = 0.004) and passage 3 (*p* = 0.002), respectively. When the TNFα concentration reached 20 ng/mL, it represented a common threshold for chondrocytes from all three passages. The effect of TNFα on *COMP* was concentration-dependent. At a TNFα concentration of 20 ng/mL, there was generally no difference in gene expression between different passages. Interestingly, the cytokine concentration of 50 ng/mL showed a different pattern, with significantly lower *COMP* gene expression in passage 2 chondrocytes compared to passage 1 (*p* = 0.01) and passage 3 (*p* = 0.014).

*Aggrecan*, another proteoglycan and a major protein component of the ECM, was affected by cytokine stimulation (Figure 1c). At a TNFα concentration of 10 ng/mL, chondrocytes from all three passages showed a strong decrease in bovine chondrocyte aggrecan expression. Interestingly, aggrecan expression showed a stronger effect at 10 ng/mL of TNFα compared to higher concentrations. There were no significant differences in cytokine effects between passages.

*Collagen 1* (*Col1*), a dedifferentiation marker, was regulated by TNFα at the gene expression level of bovine chondrocytes. The threshold for *Col1* to induce a significant TNFα-related effect was 10 ng/mL and showed a strong concentration-dependent pattern (Figure 1d). No significant differences were observed between passages.

### 2.2. Catabolic Markers

**Figure 2:**
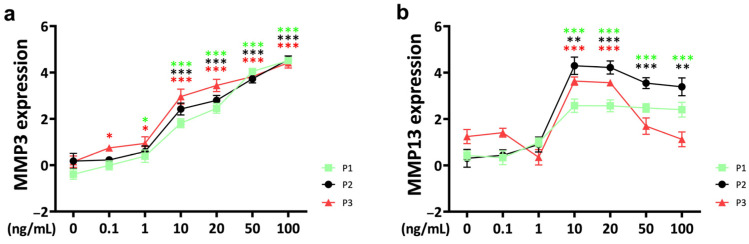
Gene expression of catabolic markers matrix metalloproteinases (**a**) *MMP3* and (**b**) *MMP13* from different passages treated with different doses (0.1 ng/mL, 1 ng/mL, 10 ng/mL, 20 ng/mL, 50 ng/mL, and 100 ng/mL) of recombinant bovine TNFα for 48 h. Gene expression data were normalized to day 0. Results were natural log transformed. * *p* < 0.05; ** *p* < 0.01; *** *p* < 0.001. The green, black, and red asterisks represent comparisons with the control at passage 1, passage 2, and passage 3, respectively. Figures show only results compared within the same passage. P1: passage 1; P2: passage 2; P3: passage 3; *MMP3*: *matrix metalloproteinase 3*; *MMP13*: *matrix metalloproteinase 13*.

*Matrix metalloproteinase 3* (*MMP3*) expression in response to TNFα supplementation was upregulated in a concentration-dependent pattern over three passages (Figure 2a). Notably, a threshold concentration of 10 ng/mL of TNFα was observed for statistically significant effects. Furthermore, at a TNFα concentration of 0.1 ng/mL, passage 3 showed a higher sensitivity to the external stimulus with significant upregulation compared to the control, whereas passage 1 and passage 2 chondrocytes were not affected (P1: *p* = 0.190; P2: *p* = 0.681; P3: *p* = 0.015). This difference decreased with increasing TNFα concentrations up to 20 ng/mL, where a significant difference between passage 3 and passage 1 was still evident (P1 vs. P3, 20 ng/mL; *p* = 0.024).

For *matrix metalloproteinase 13* (*MMP13*), the threshold concentration for a significant cytokine response was 10 ng/mL, and it did not show a concentration-dependent pattern (Figure 2b). With increasing TNFα concentration, *MMP13* expression slightly decreased in chondrocytes from passage 1 and passage 2, but the difference remained significant compared to the control group. Only passage 3 chondrocytes showed a rapid decrease in *MMP13* expression when the TNFα concentration exceeded 20 ng/mL. However, when the concentration reached 50 ng/mL, the *MMP13* gene expression levels of passage 3 chondrocytes returned to control levels.

### 2.3. Inflammation

**Figure 3:**
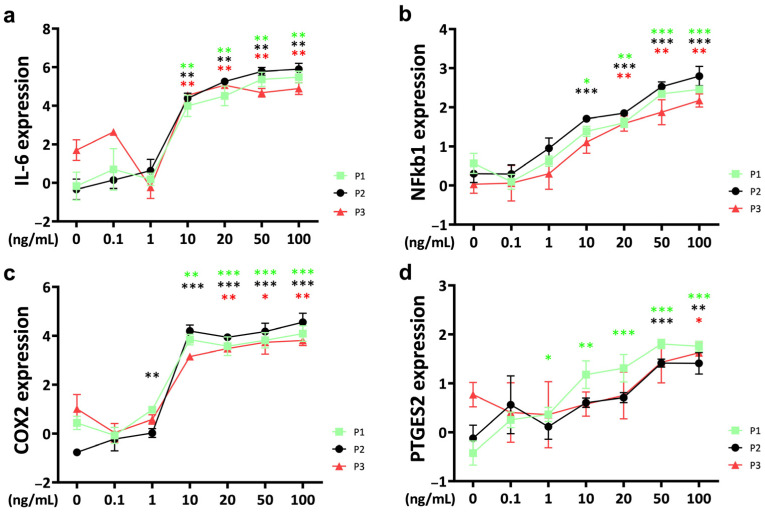
Gene expression of inflammation markers (**a**) *interleukin-6* (*IL-6*), (**b**) *nuclear factor kappa b 1* (*NFkb1*), (**c**) *cyclooxygenase-2* (*COX2*) and (**d**) *prostaglandin E synthase 2* (*PTGES2*) of bovine chondrocytes from different passages treated with different doses (0.1 ng/mL, 1 ng/mL, 10 ng/mL, 20 ng/mL, 50 ng/mL, and 100 ng/mL) of recombinant bovine TNFα for 48 h. Gene expression data were normalized to day 0. Results were natural log transformed. * *p* < 0.05; ** *p* < 0.01; *** *p* < 0.001. The green, black, and red stars represent comparisons with the control group in passage 1, passage 2, and passage 3, respectively. Figures show only results compared within the same passage. P1: passage 1; P2: passage 2; P3: passage 3; *IL-6*: *interleukin-6*; *NFkb1*: *nuclear factor kappa 1*; *COX2*: *cyclooxygenase-2*; *PTGES2*: *prostaglandin E synthase 2*.

The threshold concentration of *IL-6* for a significant TNFα-induced effect on *IL-6* gene expression in bovine chondrocytes was 10 ng/mL for all passages (Figure 3a). The upregulation of *IL-6* gene expression showed a concentration-dependent pattern. However, as the TNFα concentration continued to increase above 20 ng/mL, the rate of *IL-6* gene expression upregulation gradually decreased to saturation. Notably, at TNFα concentrations of 50 ng/mL, *IL-6* expression levels were significantly lower in passage 3 compared to passage 1 and passage 2 (P1 vs. P3: *p* = 0.043; P2 vs. P3: *p* = 0.002). Even at a higher concentration of 100 ng/mL, *IL-6* expression remained lower in passage 3 than in passage 2 (*p* = 0.024).

*Nuclear factor kappa* (*NFkB*) was upregulated by TNFα with a significant effect compared to the control at 10 ng/mL for passage 1 and passage 2 chondrocyte spheroid constructs (Figure 3b). The gene expression level of *NFkB* was lower for passage 3 chondrocytes and was significantly upregulated starting at 20 ng/mL TNFα stimulation. Gene expression of *NFkB* increased in a concentration-dependent pattern. *NFkB* expression was significantly lower in passage 3 compared to passage 2 at the highest TNFα concentration (100 ng/mL TNFα: P2 vs. P3: *p* = 0.024).

For *cyclooxygenase-2* (*COX2*)*,* the threshold concentration to induce a TNFα-related upregulation compared to the control was different between the passages, starting at 1 ng/mL for passage 2 chondrocyte spheroids, at 10 ng/mL for passage 1, and at 20 ng/mL for passage 3 constructs (Figure 3c). Concentrations higher than 20 ng/mL did not show a stronger effect. Gene expression was significantly different between passages at 10 ng/mL, with the lowest levels for passage 3 (P1 vs. P3: *p* = 0.015; P2 vs. P3: *p* = 0.006). Interestingly, *COX2* gene expression in the untreated control was different between the passages, with the lowest levels for passage 2 (P1 vs. P2: *p* = 0.004; P2 vs. P3: *p* = 0.001).

*Prostaglandin E synthase 2* (*PTGES2*) was upregulated in TNFα-stimulated chondrocytes starting at 1 ng/mL for passage 1, at 50 ng/mL for passage 2, and at 100 ng/mL for passage 3 (Figure 3d). Furthermore, the *PTGES2* gene expression level of the untreated control was significantly different between the passages (P1 vs. P3: *p* = 0.004; P2 vs. P3: *p* = 0.038). At 50 ng/mL of *PGES2*, the expression of passage 1 chondrocytes was significantly higher compared to passage 2 (P1 vs. P2: *p* = 0.041).

### 2.4. Apoptosis

**Figure 4:**
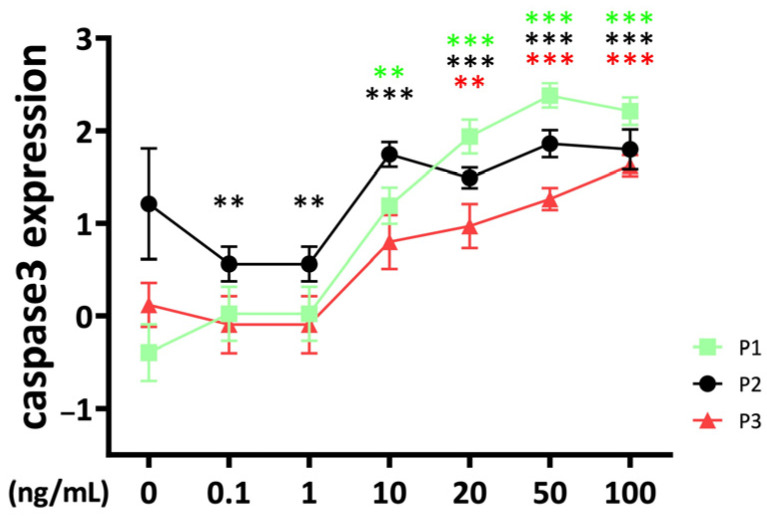
Apoptosis marker *caspase 3*, gene expression from bovine chondrocytes of different passages treated with different doses (0.1 ng/mL, 1 ng/mL, 10 ng/mL, 20 ng/mL, 50 ng/mL, and 100 ng/mL) of recombinant bovine TNFα for 48 h. Gene expression data were normalized to day 0. Results were natural log transformed. ** *p* < 0.01; *** *p* < 0.001. The green, black, and red stars represent comparisons with the control group in passage 1, passage 2, and passage 3, respectively. Figures show only results compared within the same passage. P1: passage 1; P2: passage 2; P3: passage 3.

The apoptosis marker *caspase 3* was significantly upregulated compared to the untreated control at 10 ng/mL of TNFα stimulation for passage 1 and passage 2 chondrocyte spheroid constructs (Figure 4).

The effect started in passage 3 at 20 ng/mL. Interestingly, the *caspase 3* gene expression of passage 2 constructs was lower compared to the control at low TNFα concentrations (0.1–1 ng/mL). There were significant differences between passages for TNFα concentrations higher than 10 ng/mL with a lower *caspase 3* gene expression level for passage 3 compared to passage 1 and passage 2 chondrocyte constructs (10 ng/mL: P2 vs. P3: *p* = 0.031; 20 ng/mL: P1 vs. P3: *p* = 0.014; 50 ng/mL: P1 vs. P3: *p* < 0.001; P2 vs. P3: *p* = 0.006; 100 ng/mL: P1 vs. P3: *p* = 0.019). TNFα stimulation resulted at 50 ng/mL in higher stimulation of *caspase 3* in passage 2 compared to passage 1 (P1 vs. P2: *p* = 0.014).

### 2.5. Functional Analysis of Glycosaminoglycans, Nitric Oxide, and Multiplex Inflammation Assay

**Figure 5:**
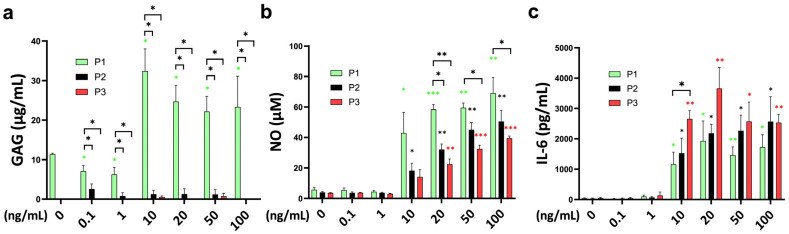
Functional analysis of the cell-related medium release of (**a**) glycosaminoglycans (GAGs; µg/mL), (**b**) nitric oxide (NO; µM), and (**c**) interleukin-6 (IL-6; pg/mL) from bovine chondrocytes from different passages treated with different doses (0.1 ng/mL, 1 ng/mL, 10 ng/mL, 20 ng/mL, 50 ng/mL, and 100 ng/mL) of recombinant bovine TNFα for 48 h. * *p* < 0.05; ** *p* < 0.01; *** *p* < 0.001. The green, black, and red stars represent comparisons with the control group in passage 1, passage 2, and passage 3, respectively. P1: passage 1; P2: passage 2; P3: passage 3.

The release of sulfated glycosaminoglycans (sGAG) into the medium was significantly different between the passages, with the highest at passage 1 (Figure 5a). TNFα increased the GAG release of passage 1 chondrocyte spheroids from 10–100 ng/mL without significant differences between the concentrations. Interestingly, the GAG concentration was slightly lower at 0.1 and 1 ng/mL of TNFα stimulation.

Nitric oxide concentration was significantly higher after TNFα supplementation starting at 10 ng/mL for passage 1 and passage 2 chondrocyte spheroid constructs compared to the untreated control (Figure 5b). A total of 20 ng/mL of TNFα was necessary to induce a significant NO release of passage 3 chondrocytes. The sensitivity to TNFα stimulation was different between the passages, with the highest NO stimulation for passage 1, followed by passage 2 and passage 3 at 20 ng/mL (P1 vs. P2: *p* = 0.006; P1 vs. P3: *p* = 0.001). The stronger NO release of passage 1 chondrocyte spheroids compared to passage 3 was also significant for 50 ng/mL and 100 ng/mL of TNFα.

Interleukin-6 (IL-6) release into the medium was in line with the gene expression data, with a strong effect starting at 10 ng/mL of TNFα for all passages (Figure 5c). The IL-6 concentration was higher compared to the control, from 10 ng/mL to 100 ng/mL in all groups. TNFα stimulation of passage 3 chondrocytes resulted in a significantly higher IL-6 release compared to passage 1 chondrocyte spheroids (P1 vs. P3: *p* = 0.036). The difference between passages was diminished at higher concentrations. Monocyte chemoattractant protein-1 (MCP-1) showed higher concentrations at 10–100 ng/mL of TNFα stimulation for all passages compared to the control (Supplementary Figure S1a). However, these differences did not reach statistical significance. Furthermore, TNFα supplementation did not influence chondrocyte viability (Supplementary Figure S1b). The analyzed markers interleukin (IL)-1alpha, interleukin (IL)-1ß, interleukin (IL)-4, interleukin (IL)-8, interleukin (IL)-10, interleukin (IL)-17a, macrophage inflammatory protein (MIP)1α, macrophage inflammatory protein (MIP)1ß, and interferon (IFN)-gamma were under the detection limit.

## 3. Discussion

In this study, a short-term systematic evaluation of the TNFα chondrocyte inflammation model was performed. In general, 48 h of TNFα supplementation caused a decrease in anabolic markers and an increase in the expression of catabolic and inflammatory markers in bovine chondrocytes. A total of 10 ng/mL was an effective concentration threshold for most of the analyzed markers. The gene expression levels of *COMP*, *aggrecan*, *Col1*, *MMP3,* and *NFkB* showed a TNFα concentration-dependent pattern. Expression profiles of selected genes, such as *Col2*, *MMP13*, *IL-6*, *NFkB*, *COX2*, and *PTGES2*, showed that expression varied between passages. The differences were more pronounced at higher cytokine concentrations. At the protein level, inflammatory effects were significant at 10 ng/mL of TNFα stimulation and showed differences between passages. Passage 3 chondrocytes showed a higher release of IL-6 into the medium compared to the other passages, whereas NO production was higher in passage 1 chondrocyte spheroid constructs.

TNFα is a pro-inflammatory cytokine that is critical in OA and stimulates various cell types involved in inflammation. This response can induce cartilage degradation. Increased, persistent inflammation leads to pain and dysfunction of the specific joint. Due to its pivotal role in OA pathogenesis, TNFα is well known as a marker of osteoarthritic progression and is often used in in vitro chondrocyte inflammation models [26]. There are several models used for OA research, with in vitro models such as the chondrocyte pellet model being favored due to their simplicity, reproducibility, and cost-effectiveness compared to in vivo models [18,27]. The chondrocyte pellet model provides a 3D environment conducive to chondrocyte differentiation and extracellular matrix synthesis [15,16]. This model simulates a more physiological environment as opposed to 2D monolayer culture, where chondrocytes proliferate and dedifferentiate over time.

According to the results of this experiment, a concentration of 10 ng/mL of TNFα effectively induced an inflammatory response in the 3D chondrocyte pellet inflammation model, which is similar to the effective dose of IL-1ß. Furthermore, the passage used played an important role, especially for the inflammation markers. Interestingly, different genes responded differently to TNFα stimulation. For example, *collagen 2* expression reached a plateau after the TNFα threshold, whereas *collagen 1* gene expression showed a concentration-dependent response. It is important to consider that collagen 2, the predominant collagen of hyaline cartilage, plays a critical role in the physiological properties of the extracellular matrix, whereas collagen 1 is a marker of dedifferentiation of expanded chondrocytes and is a minor component of the ECM in hyaline cartilage [28]. This suggests that even when chondrocytes are stimulated with TNFα at significantly different concentrations, their *collagen 2* expression levels may ultimately be similar, but the expression levels of other genes may vary significantly. These results are consistent with previous studies. Mohanraj et al. [13] investigated the response of TNFα and IL-1ß on bovine chondrocytes derived from mesenchymal stromal cells (MSCs) and native chondrocytes in agarose constructs over 6 days of culture. They found concentration-dependent GAG loss, impaired mechanical properties, and activation of MMPs upon exposure to 1–10 ng/mL of TNFα. Immunohistochemistry of collagen 2 showed reduced retention at 10 ng/mL for both MSC-derived and native chondrocytes. Interestingly, the sensitivity to external cytokine stimulation was higher for the MSC-derived chondrocytes compared to the native chondrocytes. Tilwani et al. [29] reported a concentration-dependent effect of TNFα (0.1–100 ng/mL) in a bovine 3D chondrocyte-agarose model. NO, PGE2, and MMP activity were induced, and *MMP13* and *ADAMTS-5* gene expression were significantly upregulated. This is in line with our findings. In addition, the influence of oxygen tension on chondrocytes under mechanical stimulation was analyzed. Low oxygen tension stimulated the cytokine response, whereas mechanical stimulation counteracted it.

In this experiment, we observed that the passage could influence gene expression for specific genes. Interestingly, inflammation markers (*IL-6*, *NFkB*, *COX2*, *PTGES2*, and NO) showed significant differences between passages on gene expression and protein level. Therefore, it is necessary to specify the passage in order to compare different studies. According to our results, we observed that chondrocytes from passage 3 were most sensitive to higher concentrations of TNFα (10–100 ng/mL) compared to other passages for the anabolic marker *collagen 2*. For the catabolic marker *MMP13*, chondrocytes from passage 2 were more sensitive. At lower concentrations (0.1–20 ng/mL), chondrocytes from passage 3 were more responsive than those from the other passages in terms of *MMP3* expression. However, for *MMP13*, chondrocytes from passage 2 showed increased sensitivity at concentrations ranging from 10 ng/mL to 20 ng/mL. The results are consistent with previous findings [30]. Gene expression of the inflammation marker COX2 was lower in passage 3 compared to the other passages. At the protein level, IL-6 release of passage 3 chondrocyte spheroids was higher in passage 3 for 10 ng/mL of TNFα stimulation. These different effects between passages could be a result of the detected lower NFkB expression, which might be a result of a lower TNFα receptor affinity. The transcription factor NFkB is discussed as an important inflammatory regulator that induces pro-inflammatory genes, including cytokines and chemokines [31]. Interestingly, NO levels were higher in passage 1 compared to passages 2 and 3. However, NO is controversially discussed as a pro- and anti-inflammatory marker [32]. TNFα is reported as an inducer of apoptosis [33]. Schuerwegh et al. [34] investigated the influence of TNFα at a concentration of 0.1–100 ng/mL on bovine chondrocytes in monolayer culture over 72 h. They detected a concentration-dependent NO production and apoptosis rate in a TUNEL assay.

In our study, gene expression levels of *caspase 3* as an apoptosis marker were significantly upregulated in TNFα-treated chondrocytes with a threshold concentration of 10 ng/mL. Interestingly, there was a cell culture-adapted effect in that passage 3 chondrocytes showed lower *caspase 3* expression compared to the other passages. However, the gene expression data did not result in differences in cell viability between the different treatment groups. This might be related to the short TNFα treatment of 48 h.

Bovine passage 3 chondrocytes showed a higher sensitivity to external TNFα stimulation (20 ng/mL) compared to native chondrocytes (P0) in a 3D spheroid model. The weakest response of the proinflammatory cytokine was detected in cartilage chip culture, which was the third comparison group in this study. However, this study did not compare passage 1 and passage 2 chondrocyte spheroid constructs.

Wang et al. [35] reported differences in the functional phenotype of passaged and non-passaged chondrocytes in a comparative study of 3D chondrocyte culture. Primary chondrocytes were more effective at producing hyaline-like tissue and showed less evidence of dedifferentiation compared to passage 3 chondrocytes by mechanical stimulation in a knee joint-specific bioreactor.

Obtaining sufficient cartilage tissue from small animals is challenging, often requiring multiple passages to obtain sufficient numbers of chondrocytes. The development of these models is time-consuming and often results in increased variability, leading to increased costs due to prolonged animal housing and the need for larger sample sizes to ensure statistical power in studies [16,36,37]. Although human cartilage is larger, it is typically challenging to obtain as most available cartilage comes from end-stage OA patients with limited access to healthy cartilage tissue. This leads to high variability in the specific functional phenotype and makes standardization of the inflammation model difficult. Taking into account patient-specific determinants and comorbidities, factors such as chondrocyte vitality may differ between these sources.

Cell expansion is clinically relevant in regenerative medicine. Autologous chondrocyte implantation is a surgical procedure for the treatment of focal defects with a good clinical and radiographic long-term outcome [38]. In a two-step procedure, cartilage is harvested from non-weight-bearing regions, expanded, and reimplanted in a second surgery with a 3D matrix or hydrogel [39]. However, this procedure is often performed in an osteoarthritic environment with elevated levels of proinflammatory cytokines [40], and persistent inflammation is associated with higher failure rates of cartilage repair methods [41]. For the functional phenotype and the sensitivity to inflammation, it might be important to avoid long cell expansion processes.

Our observations were based on short-term TNFα stimulation. Under normal physiological conditions, although the TNFα concentration may be low, the stimulation period is relatively longer. However, this study focused on a standardized short-term model, which is frequently used in the research field. So far, however, the early phase of this model has not been investigated in great detail by other researchers. Therefore, the idea was to provide the scientific community with a detailed analysis of the dynamics in the early phase, investigating the effects of chondrocyte passaging and TNFα concentration. This is of significant relevance to the community as it allows comparability and correct interpretation of studies with different chondrocyte passages and TNFα concentrations. Further long-term experiments with the evaluation of the time-dependent effects of TNFα are needed to validate our experimental findings.

## 4. Materials and Methods

### 4.1. Experimental Design

**Figure 6:**
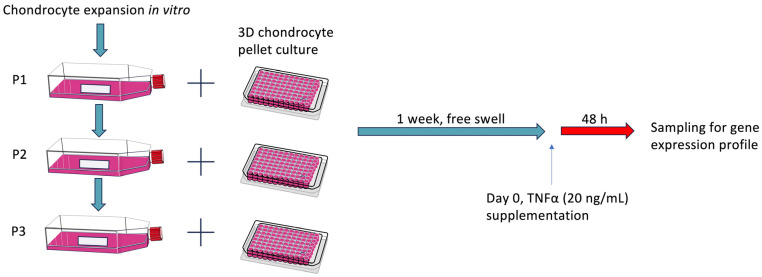
Schematic of the experimental design. Chondrocytes were expanded in vitro and then transferred into a 3D pellet culture for chondrogenic differentiation for one week. After stimulation with different concentrations of bovine recombinant TNFα for 48 h, chondrocyte pellets were collected for RT-PCR analysis. P0: passage 0; P1: passage 1; P2: passage 2; P3: passage 3. Day 0 referred to the first day that chondrocytes were exposed to TNFα stimulation in the experimental setting.

Bovine cartilage from 4 to 6-month-old calves was harvested from fetlock joints, followed by chondrocyte isolation and monolayer cell expansion, and finally transferred to 3D pellet culture at passages 1, 2, and 3 (Figure 6 and Supplementary Figure S2). After one week of free swelling, recombinant bovine TNFα was supplemented to the medium at 6 different concentrations (0.1–100 ng/mL) for 48 h. The experiment included 3 different donors with 3 technical replicates for comprehensive gene expression analysis.

### 4.2. Isolation and In Vitro Expansion of Chondrocytes

Articular chondrocytes were obtained from the fetlock joints of 4 to 6-month-old bovine animals slaughtered on the same day by a local butcher. These animals were euthanized for food production purposes, and the fetlock joints were discarded, resulting in no need for ethical approval. The articular cartilage was aseptically dissected into pieces 10 to 25 mm^2^ in size, pretreated with 0.1% pronase (Merck, Darmstadt, Germany) for 105 min, followed by digestion in 600 U/mL collagenase II (Worthington, Lakewood, NJ, USA) for 14 h according to a previously reported protocol [17]. Cell counting was performed with a hemocytometer, with trypan staining to verify viability.

Chondrocytes were seeded at a density of 14.3 × 10^3^ cells/cm^2^ in culture flasks supplied with chondrocyte growth medium consisting of Dulbecco’s modified Eagle’s medium (DMEM, high glucose, Thermo Fisher Scientific, Waltham, MA, USA) with 10% FBS (BIO&SELL, Feucht, Germany), 1% penicillin/streptomycin (Thermo Fisher Scientific, Waltham, MA, USA), and 1% L-glutamine (Sigma-Aldrich, St. Louis, MO, USA). The medium was changed every other day.

Cells were subcultured when they reached 80% confluence. Chondrocytes were rinsed twice with PBS to remove traces of serum and calcium. The cells were then digested first with 300 U/mg collagenase II and then with 0.5 mg/mL Trypsin-EDTA (Thermo Fisher Scientific, Waltham, MA, USA) for detachment. Part of the harvested chondrocytes was further expanded in vitro at the same density as mentioned above, and another part of the chondrocytes was used to construct a 3D chondrocyte pellet culture. This process was repeated up to passage 3 to obtain chondrocyte pellet models of three passages (passage 1–passage 3).

### 4.3. TNFα Chondrocyte Inflammation Model Setup

Chondrocytes were placed in 96-well V-bottom nonadhesive plates (Greiner, Kremsmünster, Austria). Each well contained 0.25 million chondrocytes supplemented with chondrogenic growth medium consisting of DMEM high glucose with 10% FBS, 60 μg/mL L-ascorbic acid phosphate (Sigma-Aldrich, St. Louis, MO, USA), 40 μg/mL L-proline (Sigma-Aldrich, St. Louis, MO, USA), 1% non-essential amino acids (Thermo Fisher Scientific, Waltham, MA, USA), 1% L-glutamine (Sigma-Aldrich, St. Louis, MO, USA), and 1% penicillin/streptomycin (Thermo Fisher Scientific, Waltham, MA, USA) without additional growth factors. Subsequently, the plates were centrifuged at 500× *g* for 5 min to form pellet-spheroids. They were then placed in an incubator (37 °C, 5% CO_2_, 21% O_2_) for one week. The medium was replaced every other day. After one week of incubation, the chondrocyte pellets were divided into seven groups, consisting of one control group without additives and six experimental groups with different cytokine concentrations of recombinant bovine TNFα (R&D Systems, Minneapolis, MN, USA) (0.1 ng/mL, 1 ng/mL, 10 ng/mL, 20 ng/mL, 50 ng/mL, and 100 ng/mL).

### 4.4. RNA Extraction, Reverse Transcription, and Gene Expression Analysis

Pellet samples were carefully pooled (*n* = 4) and placed in 1 mL TRI reagent (Molecular Research Center, Cincinnati, OH, USA) with 5 µL polyacryl carrier (Molecular Research Center). The pellets were subsequently homogenized for 10 min at 30 Hz in a tissue lyzer (Qiagen, Hilden, Germany). Phase separation was achieved by adding 100 µL of bromochloropropane (Sigma-Aldrich, St. Louis, MO, USA) per 1 mL of TRI reagent, followed by centrifugation. The aqueous phase was combined with 70% ethanol (Merck, Darmstadt, Germany) in the same volume. The following procedures were performed using the RNeasy MINI kit (Qiagen, Hilden, Germany), according to the manufacturer’s protocol.

Reverse transcription was performed with TaqMan^®®^ reverse transcription reagents (Thermo Fisher Scientific, Waltham, MA, USA) using 1 μg total RNA plus random hexamers (Thermo Fisher Scientific, Waltham, MA, USA) as primers to generate cDNA. Gene expression was analyzed using a real-time PCR system (Thermo Fisher Scientific, Waltham, MA, USA) using the TaqMan master mix with primers and probes (Thermo Fisher Scientific, Waltham, MA, USA), which are defined bovine sequences from GenBank, including the anabolic markers *collagen 2* (*Col2*), *aggrecan*, *cartilage oligomeric protein* (*COMP*), *collagen 1* (*Col1*)*;* the catabolic markers *matrix metalloproteinases 3* (*MMP3*), *matrix metalloproteinases 13* (*MMP13*); the inflammation markers *interleukin-6* (*IL-6*)*, nuclear factor kappa 1* (*NFkb1*), *cyclooxygenase-2* (*COX2*), *prostaglandin E synthase 2* (*PTGES2*)*;* and the apoptosis marker *caspase 3*, as described previously [42]. In a comparative analysis, threshold cycle (CT) values were normalized to mean CT values of the housekeeping gene 18S (∆CT) and normalized to day 0 (∆∆CT) [43]. Relative mRNA expression was calculated using the 2^−∆∆CT^ method.

### 4.5. Multiplex ELISA

Cytokines and chemokines released into the cell supernatants (interleukin (IL)-6; interleukin (IL)-1alpha; interleukin (IL)-1ß; interleukin (IL)-4; interleukin (IL)-8; interleukin (IL)-10; interleukin (IL)-17a; macrophage inflammatory protein (MIP)1α; macrophage inflammatory protein (MIP)1ß; interferon (IFN)-gamma; and monocyte chemoattractant protein-1 (MCP-1)) were analyzed using the Milliplex Bovine Cytokine/Chemokine Magnetic Beads Panel 1 kit (BCYT1-33K, Millipore, Burlington, MA, USA) and ran in 384-well format as shown previously [44]. Standards and controls were prepared following the manufacturer’s instructions. In short, 6 µL of assay buffer was added to all wells, followed by 6 µL of standard, control, or supernatant samples. Then, 6 µL of bead mix was added to each well. The plate was covered with an adhesive seal and a black lid and incubated at 4 °C with 400 rpm shaking overnight. Next, the plate was placed on a magnet, and the beads were washed twice with 50 µL of wash buffer using a BioTek 405 plate washer (Thermo Fisher Scientific, Waltham, MA, USA). The beads were allowed to settle for 2 min before each wash. After the last wash, 6 µL of detection antibody was added to each well. The plate was sealed and covered with a black lid, followed by 1 h of incubation at room temperature and 400 rpm shaking. Then, 6 µL of streptavidin-PE was added to all wells (without prior wash, as per the manufacturer’s recommendation) and further incubated for 30 min. Finally, the beads were washed as previously detailed and re-suspended with 80 µL of sheath fluid and 5 min of shaking at 400 rpm. The beads were analyzed using a FlexMap 3D instrument (Luminex, Millipore, Burlington, MA, USA) set to read at least 50 beads per analyte.

### 4.6. Nitric Oxide (NO), Glycosaminoglycan (GAG), and Viability Analysis

The nitric oxide (NO) concentration of the medium after 48 h of TNFα treatment was measured by a Griess diazotization reaction assay (*N*-(1-naphthyl)ethylenediamine dihydrochloride; Sulfanilic acid) against the nitrite standard (Promega, Walldorf, Germany) in a microplate reader at a wavelength of 548 nm. Release of sulfated gycosaminoglycans was detected by a Blyscan™ assay kit (BiColor, Belfast, UK) with a 1,9-dimethyl-methylene blue dye reagent against the GAG standard with an absorbance measurement at 625 and 675 nm. A viability analysis was performed by a commercial Cell Death Detection ELISA kit (Roche, Basel, Switzerland) using mouse monoclonal antibodies directed against DNA and histones for specific determination of mono- and oligonucleosomes in the cell culture medium against a positive control.

### 4.7. Statistical Analysis

SPSS (IBM Corp. Released 2020. IBM SPSS Statistics for Windows, Version 27.0. Armonk, NY, USA: IBM Corp.) was used for statistical analysis. As the data were assumed to be normally distributed, the independent t-test was used. Significance was defined at *p* < 0.05. Graphs were generated using GraphPad Prism 8 (Version 8.0.2, GraphPad Software Inc., San Diego, CA, USA). Gene expression data were natural log transformed to avoid skewness.

## 5. Conclusions

In the TNFα chondrocyte inflammation model, TNFα has a detrimental effect on chondrocytes in spheroid culture, with a distinct threshold for inducing inflammation. While the effects of TNFα show concentration-dependent tendencies, this is not consistent for all markers. The passage shows an influence on functional phenotype and sensitivity to external TNFα stimulation. At this point, the potential effect of passage on experimental results should be considered. Further experiments are needed to investigate the effect of TNFα concentration and passage on the model under long-term stimulation.

## Data Availability

The original contributions presented in the study are included in the article/Supplementary Materials; further inquiries can be directed to the corresponding author.

## Supplementary Materials

The following supporting information can be downloaded at: https://www.mdpi.com/article/10.3390/ijms25179136/s1.
