# Supplementary material for: TNFα-Induced Inflammation Model—Evaluation of Concentration and Passage-Dependent Effects on Bovine Chondrocytes
Source: Int J Mol Sci. 2024 Aug 23;25(17):9136. doi: 10.3390/ijms25179136 (PMC11395278; doi:10.3390/ijms25179136)
Supplement: Supplementary file 1 [file ijms-25-09136-s001.zip › ijms-3155532-supplementary.pdf]

SUPPLEMENTARY MATERIAL

Supplementary Figure S1

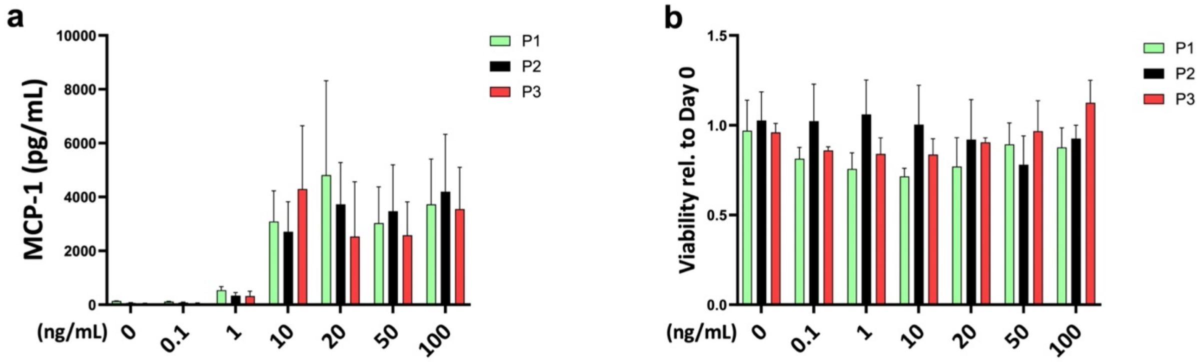

Supplementary Figure S1. Quantification of chondrocyte release of monocyte chemoattractant protein-1 (MCP-1; pg/mL) and cell viability using different passages treated with different doses (0.1 ng/mL, 1 ng/mL, 10 ng/mL, 20 ng/mL, 50 ng/mL and 100 ng/mL) of recombinant bovine TNFα for 48 hours. P1: passage 1; P2: passage 2; P3: passage 3.

Supplementary Figure S2

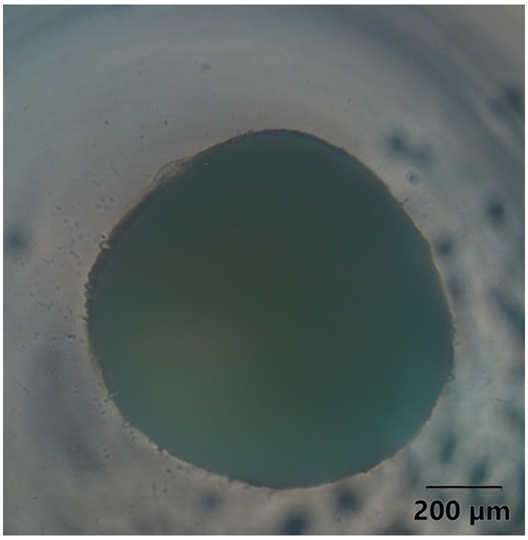

Supplementary Figure S2. Microscopy of a representative chondrocyte spheroid (passage 3).
